# Evaluation of Thermography as a Diagnostic Technique in Asymptomatic or Incipient Onychomycosis

**DOI:** 10.3390/jof9040444

**Published:** 2023-04-05

**Authors:** Julia Villar Rodríguez, Ana María Pérez Pico, Francisco Manuel García Blázquez, Juan Francisco Morán Cortés, Raquel Mayordomo Acevedo

**Affiliations:** 1 Department of Anatomy, Cellular Biology and Zoology, Centro Universitario de Plasencia, Universidad de Extremadura, 10600 Plasencia, Spain; 2 Department of Nursing, Centro Universitario de Plasencia, Universidad de Extremadura, 10600 Plasencia, Spain

**Keywords:** thermography, onychomycosis, diagnostic technique, asymptomatic onychomycosis, toenails

## Abstract

Onychomycosis is usually diagnosed symptomatically due to the very clear signs caused by the fungus on the nail surface and structure, although the growth of the infecting agent must also be verified by culture in an enriched medium. This procedure is normally lengthy (four weeks), and samples can be contaminated, delaying the prescription of appropriate and effective treatment. Only one previous study has addressed the possibility of using thermography as a diagnostic method for onychomycosis in older people (31–70 years). The present study confirms this use but in individuals aged 18–31 years with incipient mycosis and no pathological signs. Using an FLIR E60 BX camera in a study with 214 samples, we found that men had more onychomycosis than women. We observed a relation between the presence of infection and nail temperature, with a higher temperature in yeast infections (+1 °C) and a lower temperature in dermatophyte infections (−2 °C). A higher temperature by almost 1 °C was also observed in older participants. Thermography can be viewed as a new diagnostic method in asymptomatic or incipient onychomycosis, providing the thermographic camera is sufficiently sensitive and the appropriate procedure is followed, although fungal culture is always necessary to confirm recovery after treatment.

## 1. Introduction

Onychomycosis accounts for approximately 50% of all nail diseases [1,2]. This common nail infection is caught by direct contact with dermatophytes, non-dermatophyte moulds or yeasts. The nail unit is susceptible to fungal infection because it has no immunity mediated by effective cells [3]. Fungi cause changes in the colour and thickness of the nail plate, irritation, pain and, on occasion, onycholysis [4,5]. In Spain, dermatophytes are the main causal agent of onychomycosis, in particular *Trichophyton rubrum* and *T. mentagrophytes* [6,7]. Only 10% of toenail onychomycosis and 25% of fingernail onychomycosis are caused by non-dermatophyte fungi [8,9,10,11,12]. *Candida albicans* accounts for approximately 70% of onychomycosis caused by yeasts [4].

In recent decades, the prevalence of onychomycosis appears to have increased, and global prevalence is now estimated at around 5.5% [2,13,14]. The causes of this increase include population longevity, an increase in immunosuppressive treatments, a rising prevalence of obesity and greater exposure to sources of infection due to lifestyle changes, such as sports activities, communal bathing and the use of inappropriate or occlusive footwear [1,3]. Onychomycosis can occur in children but it is much more common in adults and older people, indicating that its prevalence increases with age [2,15,16].

As in other types of dermatophytosis, onychomycosis can develop without symptoms in the first stages in some anatomical regions, such as the interdigital region between the foot and the nails, and may be overlooked due to a lack of clinical signs [17]. It is important to make a correct diagnosis as early as possible in order to prescribe appropriate treatment for each case and avoid infection complications. Initial onychomycosis diagnosis is based solely on the clinical characteristics and appearance of the nails and is not always accurate. Isolating the fungus in the laboratory remains the principal diagnostic technique [18]. Traditional diagnosis is via direct observation under the microscope with potassium hydroxide (KOH) followed by fungal culture. The direct technique using KOH triggers keratin degradation and allows for visual inspection of fungal structures if they are abundant [19]. Culture allows for isolation of the causal agent of the disease and species differentiation, and it indicates whether the microorganism is active. KOH is a sensitive diagnostic test when infection is significant, but culture is much more specific and reliable [19,20].

The most sensitive diagnostic test currently available to detect the presence of mycosis in nails is polymerase chain reaction (PCR). This technique comprises in vitro amplification of a specific DNA fragment through molecular analysis using a nail fragment from which the DNA of the causal agent is extracted. Its main disadvantage is the return of false positives, as it can detect inactive, dead fungi [7,21].

Thermography is a non-invasive technique that measures the heat emitted by an object, structure or body surface, producing a pictorial representation of the surface temperature [22]. It is based on infrared radiation, a form of electromagnetic radiation with longer wavelengths than those of visible light. Any object at a temperature higher than absolute zero (i.e., T > 0 K) emits infrared radiation. Because the human eye cannot see this type of radiation, infrared measuring devices are required to acquire and process the information. Infrared measuring devices capture the infrared radiation emitted by an object and convert it into an electronic signal, obtaining a high number of pixels at the respective points that indicate temperature [23]. The final result is a false-colour image, known as a thermogram, formed by a group of pixels showing the temperature distribution of the surface [23,24].

No previous study has shown that thermography can be used to detect nails with asymptomatic or incipient onychomycosis. Its possible application in the early detection of this disease is a major discovery and an advance in diagnosis that could promote the use of appropriate treatments before the nail deteriorates, allowing for a shorter recovery time. The objective of this work is to use thermography to define the standard foot temperature in young people and screen this population to determine whether this technology could be used as a new diagnostic technique in asymptomatic or incipient onychomycosis.

## 2. Materials and Methods

Data were collected from 214 feet (107 participants; right and left foot) of people aged 18–31 years, with a slightly higher number of young participants, giving a mean age of 21.2 years (±2.80 years), with 25% older than 22 years. Approximately two-thirds of participants were women (69.2% women; 30.8% men). All participants were randomly invited to take part in the study, and all of them signed an informed consent form after the university ethics committee had approved the study. The study lasted three months, from sample collection and thermographic imaging to reading of the data obtained in the cultures, analysis of the results and report writing.

Inclusion criteria required participants to be students on the podiatry degree at the University Centre of Plasencia, University of Extremadura (Spain), and on the day the images were acquired, they had to have clean feet with no cosmetic or pharmacological substance on feet or nails, not be diagnosed with onychomycosis before participation and have normal nail morphology with no morphological alterations, thickenings or deformities and no visible signs compatible with onychomycosis. Prospective participants who did not meet the inclusion criteria or had any condition that would compromise their health status and affect the foot, such as circulatory or other systemic disorders, were excluded from the study.

The study was approved by the Bioethics Committee of the University of Extremadura, Ref. 55/2017, and conducted following the Declaration of Helsinki in accordance with current legislation on biomedical research (Law 14/2007, of 3 June).

### 2.1. Acquisition of Thermography Images

On entering the podiatry treatment room, participants were asked to keep their feet uncovered for 5 min to avoid the influence of footwear type and the state of their feet on arrival, to adapt to ambient conditions. Room temperature was kept at 25 ± 1 °C as far as possible. When participants had adjusted, they were asked to sit in the necessary position for image acquisition, with the cameras focused on their feet. Images were taken 20 cm from both naked feet after they had been positioned on a black cardboard template to avoid direct contact with the floor and prevent excessive heat loss. To document the procedure, a digital image was taken of both feet using a Xiaomi Redmi Note 9 Pro mobile phone where necessary to check for clinical signs in the nail.

The thermographic images were taken using an FLIR E60 BX camera (FLIR Systems AB, Inc., Wilsonville, OR, USA), which has thermal sensitivity of <0.045 °C and accuracy of ±2 °C and/or ±2% at room temperature. An FLIR MR77 thermo-hygrometer (Teledyne FLIR LLC, Wilsonville, OR, USA) was also used. Room temperature for the camera was 25 °C, with 45% humidity (average humidity in the treatment room). Emissivity of both devices was 0.98 (emissivity of the black body used as a reference). The camera was set to an 8-bit iron colour scale, where the dark levels indicate the lowest energies, and manually focused to achieve a sharp, well-focused image 20 cm from the participant’s feet. This distance was chosen to ensure the image allowed for visualisation of all toes and toenails, which occupied most of the image. A conventional photo was also taken.

### 2.2. Procedure in the Microbiological Analysis Laboratory

After the thermographic images and conventional photos had been acquired, a sample from the nail of the first toe was taken following the procedure used in any microbiological analysis laboratory. Sampling was conducted following the protocol described elsewhere for effective sample taking in onychomycosis [25]. The nail was disinfected with 70° alcohol, and a sample was taken using sterile nail trimmers. The sample was placed in a Petri dish for culture and analysed via direct microscopy. The nail material for the fungal culture was inoculated in Sabouraud peptone–glucose agar with cycloheximide and chloramphenicol for 2–4 weeks. When a culture showed a positive result, it was repeated for confirmation. The results were, thus, confirmed by double culture, and cases that showed a negative culture were excluded. It is important to take the images before sampling so that the emission conditions of the nail are not altered by the procedure.

### 2.3. Analysis of Thermographic Images

**Figure 1:**
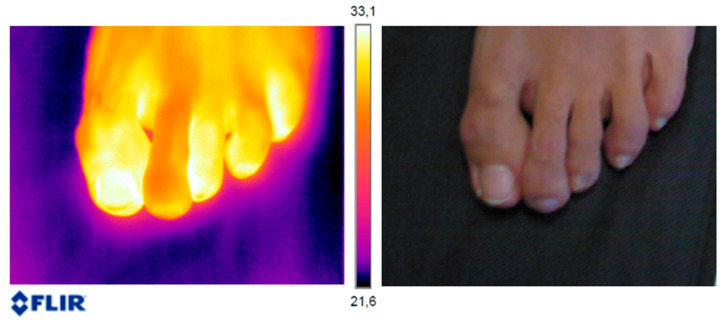
Thermographic image and conventional photo of a participant’s right foot taken with the FLIR E60 BX camera.

For the settings of the thermographic camera, it was decided that areas of lower temperature would be represented by levels of dark blue and the more whitish-yellow areas of the image would correspond to the areas with the highest temperature. The images were entered into the FLIR Tools programme for analysis. Figure 1 shows an example of the different temperatures found in a foot.

**Figure 2:**
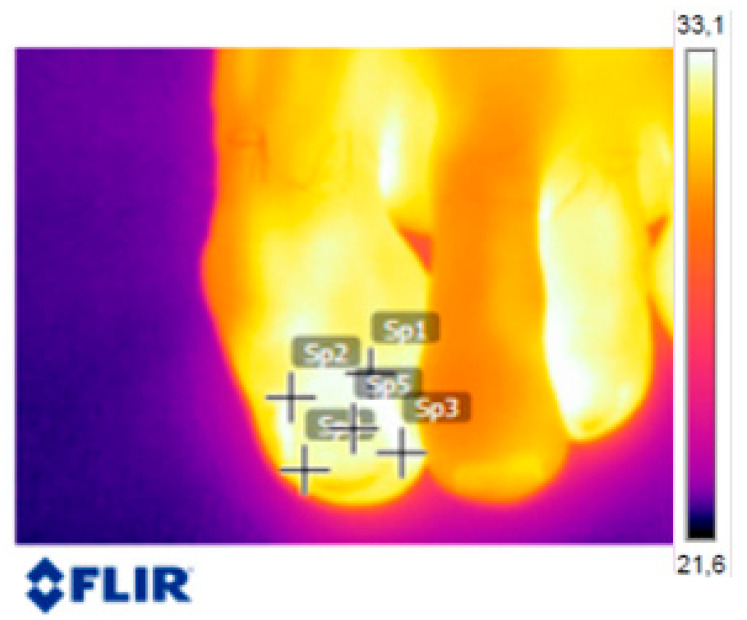
Reference points on the nail.

Five reference points on the nail plate were determined on each thermographic image for each participant (right and left foot), and all the data were recorded in a spreadsheet. Figure 2 shows these points:

- P1: proximal lateral upper point
- P2: proximal medial upper point
- P3: distal lateral lower point
- P4: distal medial lower point
- P5: middle point of the nail plate

### 2.4. Description of Variables

The variables used for the study were:

- Age: participant’s age in years
- Sex: participant’s sex
- Temperature: temperature taken at the reference points on the nail plate
- Infecting organism:
  ○ Fungi: total dermatophyte and yeast infections
  ○ Dermatophytes: dermatophyte infections
  ○ Yeasts: yeast infections

### 2.5. Statistical Analysis

Statistical analysis was performed using IBM-SPSS Statistics version 25 (reference: IBM Corp. Released 2017. IBM SPSS Statistics v 25.0 for Windows; Armonk, NY, USA).

The qualitative variables were described with frequency tables and percentages. Contingency tables were used to study the relation between the variables.

The quantitative variables were examined primarily to determine their fit to a Gaussian curve, using (a) normal Q–Q plots, (b) indices of skewness and kurtosis and (c) Kolmogorov–Smirnov goodness-of-fit test, where only a very serious deviation (*p* < 0.01) would indicate that the variable is not normally distributed.

The quantitative variables were described using the habitual tools of (a) centrality: mean and median; and (b) variability: range observed, standard deviation and interquartile range.

For comparison of means (independent subjects), the tests used were Student T-test when the distribution of the variables was normal and the non-parametric alternative (Mann–Whitney test) when the distribution was not normal.

The Chi-square test of independence was used to determine the relationship between two categorical variables. Although this test determines the existence/absence of a relation between categorial variables, the existence of a relation can be used to infer significant differences in the response variable between the categories of the explanatory factor using the values of the adjusted standardised residuals (similar to the Z values of the normal distribution, where the significance indicator shows that the residuals are ≥2).

Effect size was also calculated to express the magnitude of the differences between samples. It was expressed in R2 (scale: 0–1) to compare the different types of data collected in the variables and the different types of statistical tests and studies. To compare means, R2 was calculated using Cohen’s d. When the variables were categorical, R2 was calculated from Cramer’s V coefficient, which is similar to Pearson’s coefficient but specific for these data.

In all the inferential statistical tests, the significance value was *p* < 0.05 (level of confidence 5%) and high significance was *p* < 0.01 (level of confidence 1%).

## 3. Results

### 3.1. Presence of Infection by Location on the Nail, Sex and Age

Statistical analysis of the data obtained from the thermography images shows the presence of fungi in 14% (30) first toenails, with a slightly higher frequency for the presence of yeasts (8.9%; 19) than dermatophytes (5.1%; 11) in these nails.

Comparison of the presence of fungi between nails on the left and right foot (Table 1) shows a similar prevalence, with no significant differences (*p* > 0.05; almost no effect).

Prevalence of infection by sex was also compared (Table 1), revealing a very significant difference (*p* < 0.01), with a moderate effect size and a more frequent prevalence in men (24.2% vs. 9.5%). This result is due to a very significant difference (*p* < 0.01) with a moderate effect size in the prevalence of dermatophytes (13.6% vs. 1.4%). However, although the prevalence of yeasts was slightly higher in men (10.6% vs. 8.1%), the difference was not significant (*p* > 0.05) and the effect size did not indicate a possible relation.

Comparison of the mean age of participants with a positive diagnosis (for either infection) and a negative diagnosis showed very similar mean ages (Table 1) and, therefore, the effect of the age of the sample on the prevalence of infection was ruled out (in a population aged 18–31 years).

### 3.2. Thermography by Location on the Nail, Sex and Age

**Figure 3:**
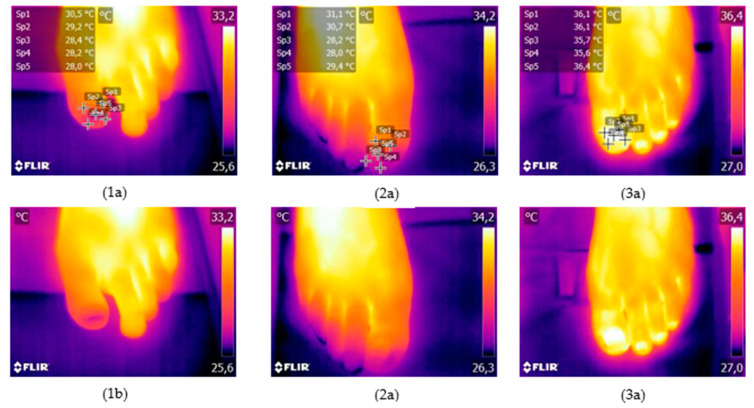
Non-infected nail (**1a**,**1b**), nail infected by dermatophytes (**2a**,**2b**) and yeast (**3a**,**3b**).

The temperature variable data taken at the five reference points were examine (example at Figure 3), as well as the mean temperature variable obtained from these data. The values of the descriptive statistics of these six variables were obtained for all data (Table 2).

The means of the five thermography points were similar, although some variability was observed within each variable among participants (range in temperature difference up to 18 °C).

Mean temperatures were compared by first toenail (right or left), sex and age. Comparison of the first toenail of the right and left foot and between men and women (Table 3) showed almost no differences between the groups, indicating that the differences were not statistically significant (*p* > 0.05) and these variables had no effect on temperature.

For comparison with age, the sample was divided into two groups by the median. The high number of participants with the median age meant that the number of cases was uneven between the two groups. The data show a small trend to a higher temperature by almost 1 °C (0.8 °C in the total mean) in the older participants (Table 3). At one point (P2), this difference was statistically significant (*p* < 0.05) and, at others, it was almost significant (*p* < 0.10). However, the effect size was always small (0.012–1.2% and 0.021–2.1%) and, therefore, there is insufficient evidence to indicate that age is a differentiating factor, although this could be examined in further studies with a sample that includes older ages than those in this study (18–31 years).

### 3.3. Relation between Thermography and Prevalence of Infection

Due to the clear difference in prevalence of infection between men and women (mainly for dermatophytes; see Table 1), we studied the possible relation between the temperatures obtained by thermography and prevalence of infection by segments according to this covariable. We analysed the 148 women first and then the 66 men, in both cases applying the comparison of means and effect size to study this possible relation. The *p*-values were estimated at 1 °C, with the expectation of finding a difference between positive diagnoses.

For the women, analysis using the variable of fungi, in general, always showed that the means of positive cases (n = 14) tend to be slightly higher than the means of negative cases (Table 4). At some points, the difference is almost 1 °C, but at others, it is only a few decimals, with a difference of 0.4 °C in the mean temperature. None of these differences were statistically significant (*p* > 0.05), by a wide margin (*p* > 0.20). However, the values of the effect sizes accompanying the comparison of means (0.021 to 0.048; almost 5% in the latter case) can mostly be considered moderate and may indicate something that was not detected due to the small number of positive cases.

Separate analysis of dermatophyte infection (Table 4) showed that temperature was clearly lower in positive (n = 2) than in negative cases. Although the small number requires us to treat this result with caution, the differences are, in general, almost significant (*p* < 0.10) and significant at two measuring points on the nail (*p* < 0.05). Comparison of positive cases due to yeast infection (n = 12) similarly showed a higher mean temperature in positive than in negative cases, with differences close to 1.5 °C at some points and a mean temperature higher by 1.3 °C (Table 4). These differences are not statistically significant (*p* > 0.05) but are almost significant (*p* < 0.10) at one measuring point. The results appear to indicate a clear trend, but the effect sizes are not large and must be classified as small. That is, if there is a difference, it is small.

For the men, analysis of total fungi (Table 5) showed similar mean temperatures in positive (n = 16) and negative cases, with no statistical significance (*p* > 0.05), by a wide margin (*p* > 0.30), and almost no effect. However, analysis of the relation between the thermography variables and dermatophyte infection (Table 5) (n = 9) shows an obviously lower temperature in the men (as in the women), at some points close to 2 °C, for a mean of almost 31 °C. All these differences are almost statistically significant (*p* < 0.10) and, at one point (P3), they are significant (*p* < 0.05). The effect sizes are mostly moderate (0.036 to 0.038), except at one point (P4), where the effect size is small (0.027). These results clearly indicate a relationship between dermatophyte infection and lower foot temperature, as observed in the women.

A comparison of cases of yeast infection in the men (Table 5) (n = 7) shows a trend towards a higher temperature (as for the women) in positive cases by almost 2 °C at some points, with the total mean temperature higher by 1.6 °C. The differences are not statistically significant (*p* > 0.05) but are not far from significance (*p* < 0.20), and at one point, the difference is almost significant (*p* < 0.10). The effect sizes are small in general (0.016 to 0.030), although some are close to moderate. These data indicate a certain trend towards a higher temperature.

Because the results differed by type of fungus but were similar between sexes, we compared the means of the thermography variables of all participants, regardless of sex, by type of infection.

*Dermatophytes* (n = 11). The results of the combined analysis (Table 6) showed statistically significant differences (*p* ≤ 0.05) in all points and in the total mean. Although the effect sizes are always small (around 0.016), the data showed a difference, as temperature is lower (by around 2 °C) in cases with dermatophyte infection. Moreover, variability within the positive group is around 1 °C higher than in the negative group, indicating greater differences among individuals for this infection.

*Yeasts* (n = 19). The results of the combined analysis (Table 6) also show significant differences (*p* < 0.05) at some points and in general, as seen in the total mean. Again, the effect sizes are always small (around 0.014) but the results indicate a difference of a higher temperature (by around 1.5 °C) in cases with a yeast infection. At the same time, unlike the other type of fungus, variability within the positive group is around 1 °C lower than in the negative group, indicating lower differences in temperature among individuals with yeast infection.

## 4. Discussion

Thermography has many advantages. It is contactless, provides two-dimensional images, can be performed quickly in real time and has no harmful radiation effects. The main advantage is that it is a non-invasive technique that does not affect or negatively interfere with measuring [23]. Because of this, thermography has become an effective tool in a range of applications, with specific benefits in the food industry, architecture [26] and veterinary medicine [23]. In recent years, it has been increasingly applied to medical studies because it is considered a very accurate and sensitive non-invasive diagnostic method [27,28,29,30]. In podiatry, its use has been proven in certain conditions, including diabetic neuropathies [24,31,32], peripheral vascular disease [33] and alterations in the lower limb muscles of runners [34,35].

Villaseñor-Mora et al. (2013) [36] reported that thermography can be used as a diagnostic method based on analysis of thermographic images, with a possible application in detecting nails with onychomycosis, but their procedure did not distinguish between dermatophytes and candida. It has, however, been used to distinguish symptomatic onychomycosis and healthy nails in diabetic patients [36].

Various authors have reported that dermatophyte fungi are the causal agents of onychomycosis, and men are more susceptible than women to this fungal infection [5,15,37,38]. Preliminary analysis of the results presented here agrees with these studies, which show that, in the age range analysed, men have proportionally more infected nails.

Analysis of the results also shows a relation between the presence of infection and foot temperature in participants aged 18–31 years with asymptomatic or incipient onychomycosis, revealing a lower temperature by almost 2 °C in positive cases of onychomycosis due to dermatophyte fungi, both in men and women, partially agreeing with the findings of Villaseñor-Mora et al. (2013) [36] and Miura et al. (2014) [39]. The former reported a lower temperature in nails infected by onychomycosis when the causal agent is a dermatophyte fungus in patients aged 30–75 years with type II diabetes. We found the same result but for an age range not analysed by these authors, thus extending their results and reinforcing the possibility of using thermography as an accurate and reliable diagnostic technique. The latter similarly observed a lower temperature in patients with onychomycosis and subungual hyperkeratosis in a population aged 59–100 years. Their study addressed patients with clear signs of onychomycosis but not with asymptomatic onychomycosis, as in the present study. Different infecting agents cause different nail disorders. Yeasts usually cause paronychia (inflammation of the lateral nail fold or paronychium), which could explain the higher temperature in the area where the infection starts. Dermatophytes alter the nail plate by forming dermatophytomas (grooves in the nail plate with detritus of dead tissue), as explained by Veiga et al. (2022) [40]. This could be associated with the lower temperature when dermatophytes are the infecting agent, although more studies are needed to confirm this hypothesis.

No previous study has addressed the relation between the presence of mycotic infection with yeast as the causal agent and nail temperature. We identified a higher temperature, by 1.5 °C, in positive cases, both in men and women. This indicates the previously unstudied difference in behaviour and effect of infections depending on the type of infecting fungus.

The present study rules out an effect of age on the prevalence of infection in the age range of the population studied (18–31 years). In a 2016 Canadian study, Gupta et al. [5] reported that onychomycosis is related to age, with a prevalence of 0.4−2% for individuals aged 18–30 years and 24% for persons aged more than 80 years. The low prevalence in our study may be the result of studying a young, similarly aged university population with no symptoms.

Miura et al. (2014) [39] did not report a significant correlation between temperature and age. Our study, however, showed a higher temperature in older participants without infection (25–30 years) by almost 1 °C.

One limit of this study is that a very sensitive, highly accurate thermographer is needed to use this technique for diagnosis, requiring considerable investment. The statistical interpretation is also a limitation because our results show almost significant values (*p* < 0.1), although analysis of the effect size (ES) indicates that thermography is a valid technology in these proven conditions. However, the study should be extended to include a larger sample size. Asymptomatic onychomycosis is not easy to detect, and a very large sample must be studied with a high number of participants for measurements. Despite the many novel and promising results obtained in this work, more studies are needed to enlarge the sample, both for a greater age range and in older ages, and to determine the behaviour of temperature in asymptomatic or incipient onychomycosis infection.

## 5. Conclusions

Our study shows that sex affects the prevalence of dermatophytes, as men are more affected than women by this infection.

The study also indicates a relation between the presence of infection and nail temperature, as temperature is lower in dermatophyte infection by around 2 °C and higher in yeast infection by around 1 °C, both in men and women.

A higher temperature by almost 1 °C was observed in older participants (0.8 °C total mean). However, the effect size is small, indicating that there is insufficient evidence to identify age as a differentiating factor, although this circumstance should be explored in future studies with ages higher than those in our study group (18–31 years).

The results obtained show that thermography can be viewed as a new diagnostic method for asymptomatic or incipient onychomycosis, with the advantages that it is non-invasive, helps to prevent transmission of infection, is completely innocuous, allows for a much faster response time and is inexpensive. This type of diagnostic technique will help to improve primary prevention of mycosis in the foot.

## Figures and Tables

**Table 1:** Presence of infection. Comparison between first toenail for each foot, between sexes and relation to age (mean).

		First Toenail in Each Foot				Sexes				Age (Mean)			
Infection	Total (n = 214)	Toenail		Comparison of Means		Sex		Comparison of Means		Mean Age		Comparison of Means	
		Right (n = 107)	Left (n = 107)	R^2^ E	P-V	Female (n = 148)	Male (n = 66)	R^2^ E	P-V	Positive	Negative	R^2^ E	P-V
Fungi (positive)	14.0% (30)	13.1% (14)	15.0% (16)	0.001	0.694 (ns)	9.5% (14)	24.2% (16)	0.039	0.004 (**)	21.7 years (±2.5)	21.1 years (±2.9)	0.005	0.303 (ns)
Dermatophytes (positive)	5.1% (11)	4.7% (5)	5.6% (6)	0.000	0.757 (ns)	1.4% (2)	13.6% (9)	0.066	0.000 (**)	20.9 years (±2.4)	21.2 years (±2.8)	0.001	0.745 (ns)
Yeast (positive)	8.9% (19)	8.4% (9)	9.3% (10)	0.000	0.810 (ns)	8.1% (12)	10.6% (7)	0.002	0.553 (ns)	22.1 years (±2.4)	21.1 years (±2.8)	0.011	0.131 (ns)

R^2^ E = R^2^ Effect, P-V = *p*-Value, ns = not significant, ** = Highly significant.

**Table 2:** Descriptive analysis of thermography variables. N = 224.

Thermography (°C)	Centrality		Variability		Percentile		
	Mean	IC 95% of the Mean	Range (min/max)	Standard Deviation	P25	Median	P75
Temperature P-1	30.9	30.5–31.4	21.4/36.2	3.4	27.8	31.5	33.8
Temperature P-2	30.7	30.2–31.2	21.1/39.7	3.5	27.5	31.1	33.7
Temperature P-3	29.6	29.2–30.1	20.9/36.1	3.6	26.6	29.7	32.7
Temperature P-4	29.6	29.1–30.1	20.8/39.4	3.6	26.6	29.5	32.7
Temperature P-5	30.7	30.2–31.2	21.3/36.9	3.6	27.6	30.9	33.9
Mean temperature	30.3	29.8–30.8	21.1/36.1	3.5	27.3	30.5	33.4

**Table 3:** Comparison of thermography variables by first toenail on each foot, sex and age.

	First Toenail of Each Foot				Sex				Age			
Thermography (°C)	Mean (Standard Deviation)		Comparison of Means		Mean (Standard Deviation)		Comparison of Means		Mean (Standard Deviation)		Comparison of Means	
	Right Foot (n = 107)	Left Foot (n = 107)	ES	P-V	Female (n = 148)	Male (n = 66)	ES	P-V	<=21 years (n = 130)	=>22 years (n = 84)	ES	P-V
Temperature P-1	31.0 (±3.4)	30.9 (±3.4)	0.000	0.836 (ns)	30.8 (±3.5)	31.2 (±3.3)	0.004	0.368 (ns)	30.6 (±3.4)	31.4 (±3.2)	0.013	0.103 (ns)
Temperature P-2	30.7 (±3.5)	30.7 (±3.6)	0.000	0.913 (ns)	30.6 (±3.5)	31.0 (±3.6)	0.003	0.440 (ns)	30.3 (±3.5)	31.3 (±3.3)	0.021	0.034 (*)
Temperature P-3	29.5 (±3.5)	29.8 (±3.6)	0.001	0.617 (ns)	29.5 (±3.6)	29.9 (±3.4)	0.003	0.453 (ns)	29.3 (±3.6)	30.1 (±3.3)	0.012	0.107 (ns)
Temperature P-4	29.6 (±3.6)	29.7 (±3.7)	0.000	0.829 (ns)	29.6 (±3.8)	29.6 (±3.3)	0.000	0.958 (ns)	29.3 (±3.7)	30.2 (±3.4)	0.014	0.084 (ns)
Temperature P-5	30.7 (±3.6)	30.7 (±3.5)	0.000	0.995 (ns)	30.5 (±3.7)	31.0 (±3.3)	0.003	0.431 (ns)	30.3 (±3.6)	31.2 (±3.4)	0.014	0.081 (ns)
Mean temperature	30.3 (±3.5)	30.3 (±3.5)	0.000	0.932 (ns)	30.2 (±3.6)	30.5 (±3.3)	0.002	0.510 (ns)	30.0 (±3.5)	30.8 (±3.3)	0.015	0.073 (ns)

ES = effect size, ns = not significant, * = Significant.

**Table 4:** Relation between results of thermography variables and prevalence of infection in women: fungi, dermatophytes and yeast.

	Fungi				Dermatophytes				Yeast			
Thermography (°C)	Mean (Standard Deviation)		Comparison of Means		Mean (Standard Deviation)		Comparison of Means		Mean (Standard Deviation)		Comparison of Means	
	P (n = 14)	N (n = 134)	ES	P-V	P (n = 2)	N (n = 146)	ES	P-V	P (n = 12)	N (n = 136)	ES	P-V
Temperature P-1	31.0 (±2.8)	30.8 (±3.5)	0.021	0.398 (ns)	26.3 (±0.3)	30.8 (±3.4)	0.023	0.033 (*)	31.8 (±2.1)	30.7 (±3.6)	0.008	0.146 (ns)
Temperature P-2	31.1 (±2.8)	30.5 (±3.6)	0.046	0.290 (ns)	26.2 (±0.4)	30.6 (±3.6)	0.021	0.038 (*)	31.9 (±2.0)	30.5 (±3.6)	0.012	0.090 (ⴕ)
Temperature P-3	30.1 (±3.1)	29.5 (±3.7)	0.048	0.281 (ns)	25.6 (±0.4)	29.6 (±3.6)	0.016	0.060 (ⴕ)	30.8 (±2.6)	29.4 (±3.7)	0.011	0.100 (ns)
Temperature P-4	30.0 (±2.8)	29.6 (±3.9)	0.034	0.342 (ns)	26.1 (±0.4)	29.7 (±3.8)	0.012	0.089 (ⴕ)	30.7 (±2.4)	29.5 (±3.9)	0.007	0.157 (ns)
Temperature P-5	30.9 (±2.9)	30.5 (±3.7)	0.031	0.354 (ns)	26.7 (±0.2)	30.6 (±3.7)	0.016	0.065 (ⴕ)	31.6 (±2.5)	30.5 (±3.7)	0.007	0.149 (ns)
Mean temperature	30.6 (±2.8)	30.2 (±3.6)	0.037	0.329 (ns)	26.2 (±0.3)	30.3 (±3.5)	0.018	0.052 (ⴕ)	31.4 (±2.3)	30.1 (±3.6)	0.009	0.123 (ns)

P = positive, N = negative, ES = effect size, P-V = *p*-Value, ns = not significant, ⴕ = Almost significant * = Significant.

**Table 5:** Relation between results of thermography variables and prevalence of infection in men: fungi, dermatophytes and yeast.

	Fungi				Dermatophytes				Yeast			
Thermography (°C)	Mean (Standard Deviation)		Comparison of Means		Mean (Standard Deviation)		Comparison of Means		Mean (Standard Deviation)		Comparison of Means	
	P (n = 16)	N (n = 50)	ES	P-V	P (n = 9)	N (n = 57)	ES	P-V	P (n = 7)	N (n = 59)	ES	P-V
Temperature P-1	31.0 (±4.0)	31.3 (±3.1)	0.002	0.362 (ns)	29.7 (±4.6)	31.5 (±3.1)	0.036	0.064 (ⴕ)	32.7 (±2.4)	31.1 (±3.4)	0.022	0.115 (ns)
Temperature P-2	30.6 (±4.2)	31.1 (±3.4)	0.004	0.305 (ns)	29.2 (±4.9)	31.2 (±3.3)	0.037	0.060 (ⴕ)	32.3 (±2.4)	30.8 (±3.7)	0.016	0.157 (ns)
Temperature P-3	29.7 (±3.7)	30.0 (±3.3)	0.002	0.358 (ns)	28.1 (±3.8)	30.2 (±3.2)	0.045	0.044 (*)	31.6 (±2.6)	29.7 (±3.4)	0.030	0.083 (ⴕ)
Temperature P-4	26.9 (±3.6)	29.7 (±3.3)	0.000	0.446 (ns)	28.3 (±4.0)	29.9 (±3.2)	0.027	0.084 (ⴕ)	31.2 (±2.2)	29.5 (±3.4)	0.025	0.101 (ns)
Temperature P-5	30.6 (±4.0)	31.1 (±3.2)	0.003	0.328 (ns)	29.3 (±4.5)	31.2 (±3.1)	0.038	0.058 (ⴕ)	32.3 (±2.6)	30.8 (±3.4)	0.019	0.132 (ns)
Mean temperature	30.3 (±3.9)	30.6 (±3.2)	0.002	0.356 (ns)	28.9 (±4.4)	30.8 (±3.1)	0.038	0.060 (ⴕ)	32.0 (±2.4)	30.4 (±3.4)	0.023	0.112 (ns)

P = positive, N = negative, ES = effect size, P-V = *p*-Value, ns = not significant, ⴕ = Almost significant * = Significant.

**Table 6:** Relation of results of thermography variables and prevalence of dermatophyte and yeast infection.

	Dermatophytes				Yeast			
Thermography (°C)	Mean (Standard Deviation)		Comparison of Means		Mean (Standard Deviation)		Comparison of Means	
	Positive (n = 11)	Negative (n = 203)	ES	P-V	Positive (n = 19)	Negative (n = 195)	ES	P-V
Temperature P-1	29.0 (±4.3)	31.0 (±3.3)	**0.016**	0.031 (*)	32.1 (±2.2)	30.8 (±3.5)	0.012	0.056 (ⴕ)
Temperature P-2	28.7 (±4.5)	30.8 (±3.4)	**0.017**	0.027 (*)	32.0 (±2.1)	30.6 (±3.6)	**0.014**	0.043 (*)
Temperature P-3	27.7 (±3.6)	29.8 (±3.5)	**0.017**	0.029 (*)	31.1 (±2.6)	29.5 (±3.6)	**0.016**	0.031 (*)
Temperature P-4	27.9 (±3.7)	29.7 (±3.6)	**0.013**	0.050 (*)	30.9 (±2.2)	29.5 (±3.7)	0.011	0.061 (ⴕ)
Temperature P-5	28.8 (±4.2)	30.8 (±3.5)	**0.014**	0.041 (*)	31.9 (±2.5)	30.6 (±3.6)	0.011	0.064 (ⴕ)
Mean temperature	28.4 (±4.1)	30.4 (±3.4)	**0.016**	0.032 (*)	31.6 (±2.3)	30.2 (±3.6)	**0.013**	0.047 (*)

P = positive, N = negative, ES = effect size, P-V = *p*-Value, ns = not significant, ⴕ = Almost significant * = Significant.

## Data Availability

Not applicable.
